# Dynamic Strain Measurements on Automotive and Aeronautic Composite Components by Means of Embedded Fiber Bragg Grating Sensors

**DOI:** 10.3390/s151027174

**Published:** 2015-10-26

**Authors:** Alfredo Lamberti, Gabriele Chiesura, Geert Luyckx, Joris Degrieck, Markus Kaufmann, Steve Vanlanduit

**Affiliations:** 1 Department of Mechanical Engineering, Vrije Universiteit Brussel (VUB), Pleinlaan 2, Elsene 1050, Belgium; 2 Department of Material Science and Engineering, Ghent University Technologiepark Zwijn. 903, Zwijnaarde 9052, Belgium; E-Mails: gachiesu.Chiesura@UGent.be (G.C.); Geert.luyckx@ugent.be (G.L.); Joris.Degrieck@UGent.be (J.D.); 3 Sirris Leuven-Gent Composites Application Lab, Celestijnenlaan 300C, Heverlee 3001, Belgium; E-Mail: Markus.Kaufmann@sirris.be; 4 Faculty of Applied Engineering, University of Antwerp, Campus Hoboken Salesianenlaan 90, Antwerp 2660, Belgium; E-Mail: Steve.Vanlanduit@uantwerpen.be

**Keywords:** fiber Bragg grating, optical sensing, CFRP and GFRP composites, dynamic measurements, modal parameters, demodulation algorithms

## Abstract

The measurement of the internal deformations occurring in real-life composite components is a very challenging task, especially for those components that are rather difficult to access. Optical fiber sensors can overcome such a problem, since they can be embedded in the composite materials and serve as *in situ* sensors. In this article, embedded optical fiber Bragg grating (FBG) sensors are used to analyze the vibration characteristics of two real-life composite components. The first component is a carbon fiber-reinforced polymer automotive control arm; the second is a glass fiber-reinforced polymer aeronautic hinge arm. The modal parameters of both components were estimated by processing the FBG signals with two interrogation techniques: the maximum detection and fast phase correlation algorithms were employed for the demodulation of the FBG signals; the Peak-Picking and PolyMax techniques were instead used for the parameter estimation. To validate the FBG outcomes, reference measurements were performed by means of a laser Doppler vibrometer. The analysis of the results showed that the FBG sensing capabilities were enhanced when the recently-introduced fast phase correlation algorithm was combined with the state-of-the-art PolyMax estimator curve fitting method. In this case, the FBGs provided the most accurate results, *i.e.*, it was possible to fully characterize the vibration behavior of both composite components. When using more traditional interrogation algorithms (maximum detection) and modal parameter estimation techniques (Peak-Picking), some of the modes were not successfully identified.

## 1. Introduction

Fiber-reinforced polymers (FRPs) are a class of composite materials that are obtained by assembling together a reinforcing phase and a matrix phase [1,2]. The reinforcing phase consists of fibers (carbon, glass, polyaramid, natural), while the matrix can be a thermoplastic, thermoset or ceramic material. FRPs offer superior specific mechanical properties (*i.e.*, mechanical properties per unit density) than other engineering materials (such as metals). Moreover, they are resistant to corrosion and have excellent fatigue life [1,2,3]. This explains why, in the last few decades, FRPs have become extremely popular in many industrial fields [4]. In the aerospace and automotive sectors, carbon fiber-reinforced (CFRPs) and glass fiber-reinforced polymers (GFRPs) are the most widespread, since they offer the highest strength-to-weight ratios [2,4]. However, compared to their metallic counterparts, modern CFRPs and GFRPs are often over-designed, since damage mechanism are not yet well understood and cannot be easily simulated. As a result, there is often a lack of confidence in design analysis methods. For the same reason, CFRPs and GFRPs usually require more frequent inspections and monitoring. To overcome these drawbacks, smart fiber-reinforced polymer (SFRP) composites have started to be investigated. In general, the term “smart” indicates multifunctional composites that can perform functions, such as sensing stress, strain, pressure, temperature or damage. Optical fiber sensors are a very attractive proposition for deployment in SFRPs [5] since: (i) they have a small size and weight; therefore, they are suitable for being embedded inside composite preforms during manufacturing; (ii) they can be used in harsh environments where electrical-based sensors may not survive [6,7,8]; (iii) they can be employed to sense different physical measurands (such as strain, temperature, force, pressure, chemical composition, *etc.*); (iv) they are characterized by a long lifetime (more than 20 years), and they are stable over time (no calibration required); and (v) they can be used for production monitoring [9,10,11,12], as well as for structural health monitoring (SHM) [13] purposes. Among the different fiber optic sensors developed up to now, fiber Bragg grating (FBG) sensors are one of the most suitable for composite damage detection and monitoring [14,15,16]. In fact, they allow (quasi-)distributed measurement capabilities by spatially multiplexing several FBGs along the same optical fiber line. When this fiber is then interrogated with a broadband light, each FBG reflects a specific wavelength (named the Bragg wavelength), which carries the local information about the physical measurand (for instance strain). Numerous applications of FBG sensors for damage detection in composite materials have been reported in the literature [17,18,19,20,21,22,23,24,25]. However, in most of these studies, the FBG sensors have been used under static and/or quasi-static loading conditions, and the damage assessment has been based on the analysis of the measured local strain levels. The biggest shortcoming of this approach is that the damage detection capability depends on the relative position between the FBG sensor and the damage: if the FBG is too far from the damage, than it is not able to detect it. To overcome this issue, alternative SHM approaches can be used, such as those based on modal analysis. For a long time, modal analysis has been associated with the use of displacement-based sensors, such as accelerometers and laser vibrometers. However, during the last two decades, the interest in strain-based modal analysis has been constantly increasing, both in academia and in industry. Many works exist where strain modal analysis has been performed by means of strain gauges [26,27]. Unfortunately, these sensors are more difficult to use than accelerometers (due to calibration requirements, high temperature sensitivity, non-linear response) and present several limitations. FBG sensors represent a better alternative to strain gauges. The FBG capability to perform modal analysis has been investigated by different authors [28,29,30,31,32,33,34,35]. However, few works exist where embedded FBG sensors have been used to measure the modal characteristics of real-life composite structures [36,37,38]. In 2006, Cusano *et al.* [36] performed the modal analysis of the wing of an unmanned airplane model by means of FBG sensors embedded in the composite wing spar. The modal parameters they were able to retrieve ranged up to 170 Hz. For their analysis, they developed a passive detection scheme based on the combination of optical filtering and broadband light interrogation. Such an interrogation system has the benefit of being simple and cost effective. However, it does not exploit a key advantage of an FBG sensor, the fact that the information of the measurand is encoded in the reflection spectrum. The added benefits of working with full-spectrum interrogators has been recently shown in some publications [39,40]. For instance, full-spectrum interrogation is to be preferred when the embedded FBGs experience complex and multi-component stress states (as happens near damaged regions).

In this paper, we describe the capability of full-spectrum measurements of embedded FBG sensors to perform modal analysis of two real-life industrial composite components. The first component is a CFRP automotive control arm, which is part of an automotive rear wheel suspension system. The second component is a GFRP hinge arm designed for the wing leading-edge high-lift device of a modern aircraft. In the original design, such a component was meant to be made of CFRP. However, to provide a proof of concept and to contain the cost at the same time, this research was conducted on a preliminary prototype made of GFRP. Both composite components were manufactured via the resin transfer molding (RTM) technique [41]. During the manufacturing process, the CFRP control arm was instrumented with two optical fiber lines, carrying a total of 12 multiplexed FBGs; while the GFRP hinge arm was equipped with one optical fiber with three multiplexed FBGs. After demolding and post-curing, the two components were tested to retrieve their modal parameters. An electromechanical shaker was used to excite the two components with a multisine load (a multisine is a sum of harmonically-related sinusoidal signals). The internal strain levels induced by the mechanical vibrations were measured by dynamically acquiring and demodulating the full-spectrum of the embedded optical fibers. A commercially-available FBGS scan FBG 804D interrogator [42] (from FBGS) controlled by an in-house-developed MATLAB^®^ script was used for the acquisition. The spectral demodulation and the calculation of the strain time histories were carried out by using two different algorithms. The first is a conventional maximum-detection (MD) algorithm, while the second is the novel fast phase correlation (FPC) [43,44] algorithm, recently proposed by the authors. The strain time histories were then transformed to the frequency domain, and the modal parameters of each component were retrieved via two different modal parameter estimation techniques: the Peak-Picking [45] and the poly reference least-squares modal parameter estimator PolyMax [46,47]. For the sake of comparison, reference analyses were additionally conducted using a laser Doppler vibrometer (LDV) [48]. The analyses of the results showed that the best correspondence between FBG and LDV measurements was obtained using the combination FPC-Polymax. In fact, the FPC algorithm performed better than the MD, providing demodulated FBG signals with higher signal-to-noise ratios (especially in the case of distorted reflected peak). At the same time, the PolyMax estimator was able to overcome the limitation of the Peak-Picking technique, allowing the estimation of modal parameters otherwise impossible to retrieve. Compared to the combination FPC-PolyMax, the combination MD-PolyMax was less accurate and even failed in one instance. This proves that an appropriate selection of the processing algorithms enhances the FBGs’ sensing capabilities and allows them to effectively measure vibrations, even when embedded in complex real-life industrial composites. This paper is further structured as follows. Section 2 presents the general concepts regarding FBG sensors: it first recalls the FBG sensing principle and, after introducing the maximum detection (MD) and the fast phase correlation (FPC) demodulation algorithms, it deals with the application of FBGs in the framework of strain-based modal analysis. Section 3 provides the details regarding the manufacturing of the composite components and the embedding of the FBG sensors. Section 4 describes the experimental procedure and discusses the obtained results. Finally, Section 5 contains the conclusive remarks and some ideas for future developments.

## 2. Fiber Bragg Grating as Sensing Devices and Their Application for Modal Parameter Estimation

### 2.1. The FBG Working Principle

**Figure 1:**
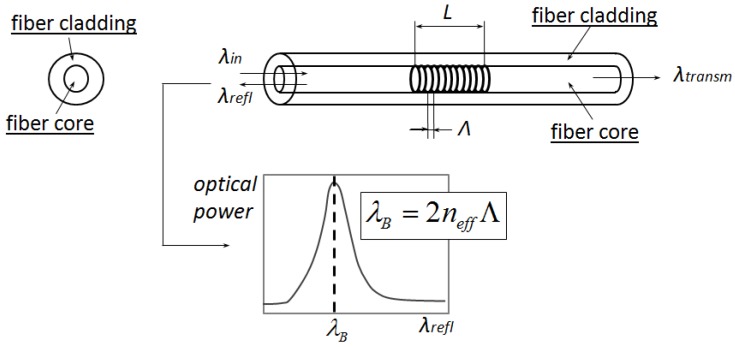
Schematic representation of a Bragg grating of length *L* and pitch Λ inscribed in an optical fiber.

A fiber Bragg grating (FBG) is an optical fiber where a grating is inscribed inside the fiber core (Figure 1). Within the grating region, the index of refraction experiences a periodic modulation, which makes the grating act as a band-pass filter.

When broadband light is injected into the fiber, the grating reflects one particular wavelength, named the Bragg wavelength. The Bragg wavelength $\lambda_{B}$ is given by:

(1) $$\lambda_{B}=2\;n_{\mathrm{eff}}\;\Lambda$$

where $n_{\mathrm{eff}}$ is the effective refractive index averaged over the entire grating length *L* and Λ indicates the grating pitch (Figure 1). Changes in the Bragg wavelength are associated with modifications of either or both $n_{\mathrm{eff}}$ and Λ. In general, when a thermomechanical load acts on the FBG, the Bragg wavelength shift $\Delta \lambda_{B}$ is given by:

(2) $$\Delta \lambda_{B}=\lambda_{B}\left(\frac{1}{n_{\mathrm{eff}}}\frac{dn_{\mathrm{eff}}}{dT}+\frac{1}{\Lambda }\frac{d\Lambda }{dT}\right)\Delta T+\lambda_{B}\left(\frac{1}{n_{\mathrm{eff}}}\frac{dn_{\mathrm{eff}}}{d\epsilon }+\frac{1}{\Lambda }\frac{d\Lambda }{d\epsilon }\right)\Delta \epsilon$$

The first term on the right-hand side of Equation (2) represents the effect of temperature on the Bragg wavelength shift, with $\frac{1}{n_{\mathrm{eff}}}\frac{dn_{\mathrm{eff}}}{dT}$ being the thermo-optic coefficient and $\frac{1}{\Lambda }\frac{d\Lambda }{dT}$ the thermal-expansion coefficient. The second term on the right-hand side of Equation (2) represents the strain contribution to the Bragg wavelength shift. It corresponds to a change in the grating periodicity and the strain-optic-induced change in the refractive index [49]. Assuming isothermal conditions and strain acting only in the fiber longitudinal direction, Equation (2) becomes:

(3) $$\Delta \lambda_{B}=\lambda_{B}\left(1-p_{\mathrm{eff}}\right)\Delta \epsilon$$

where $p_{\mathrm{eff}}=\frac{{n_{\mathrm{eff}}}^{2}}{2}\left[p_{12}-\upsilon \left(p_{11}+p_{12}\right)\right]$, $p_{11}$ and $p_{12}$ are the components of the fiber-optic strain tensor and *υ* is the Poisson’s ratio. For GeO${}_{2}$-doped (quartz) fiber, a typical value of the effective photo-elastic coefficient $p_{\mathrm{eff}}$ is 0.204 [50].

### 2.2. Demodulation of FBG Signals

The demodulation of FBG sensor signals consists of the computation of the Bragg wavelength shifts from which one reconstructs the external physical measurand acting on the optical fiber. In this research article, vibration measurements will be carried out in isothermal conditions; therefore, the only measurand that will be retrieved by means of Equation (3) is strain. This assumption is realistic, because the measurement time for vibration analyses is low (order of seconds). Clearly, the more accurate and precise the demodulation technique is, the better the estimation of the strain will be. Many demodulation algorithms have been proposed in the literature [43,44,51,52,53,54]. In this article, the traditional maximum detection (MD) algorithm and the novel fast phase correlation (FPC) [43,44] algorithm are adopted.

The MD employed throughout this article computes the Bragg wavelength shift $\Delta \lambda_{B}$ between two FBG reflected spectra R(λ) and $R^{\prime }\left(\lambda \right)=R\left(\lambda +\Delta \lambda_{B}\right)$ using the following equation:

(4) $$\Delta \lambda_{B}=\arg\max_{\lambda }\left\{{}^{p}R^{\prime }\left(\lambda \right)\right\}-\arg\max_{\lambda }\left\{{}^{p}R\left(\lambda \right)\right\}$$

where *λ* is the wavelength, while ${}^{p}R\left(\lambda \right)$ and ${}^{p}R^{\prime }\left(\lambda \right)$ indicate the spectra obtained with a *p* point quadratic interpolation around the peak wavelength of the original reflection spectra R(λ) and $R^{\prime }\left(\lambda \right)$, respectively. In this paper, p=5, which means that a sub-wavelength interpolation is performed using two values left and two values right of the point of maximum reflectivity. Differently from the MD, the FPC calculates the Bragg wavelength shift $\Delta \lambda_{B}$ through the following equation:

(5) $$\Delta \lambda_{B}=\underset{2\leq k\leq M}{\mathrm{median}}\;\left[\left(∠R^{\prime }\left(k\right)-∠R\left(k\right)\right)\frac{Nk\;\delta \lambda }{2\pi }\right],\;k=2,\ldots ,M<<N$$

**Figure 2:**
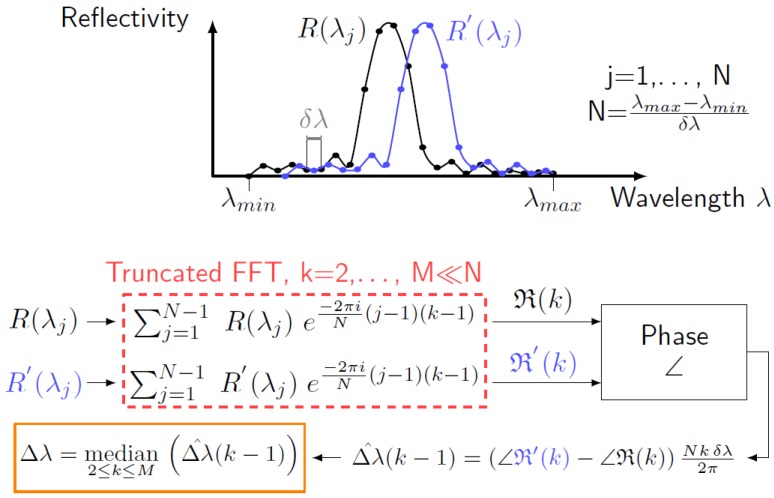
Schematic representation of the Bragg wavelength shift calculation between *R* and $R^{\prime }$ by means of the fast phase correlation (FPC) algorithm.

In Equation (5), R and $R^{\prime }$ are the Fourier transforms of *R* and $R^{\prime }$, respectively; the symbol *∠* indicates the phase of a complex number; the letter *k* is the generic Fourier spectral line, while *M* is the maximum number of Fourier spectral lines taken into account by the algorithm. *N* is the number of sampling points constituting both R(λ) and $R^{\prime }\left(\lambda \right)$ spectra, and $\delta \lambda =\lambda_{j+1}-\lambda_{j}$ is the wavelength resolution. Figure 2 provides a graphical explanation of the FPC working principle, which leads to Equation (5). For more theoretical details on the algorithm, refer to Lamberti *et al.* [43].

Compared to other demodulation algorithms (such as the MD), the FPC offers several benefits. Firstly, it produces precise and accurate results even when the wavelength resolution is poor and/or the reflected peak is noisy or partially distorted. Secondly, the FPC has a very high execution speed; therefore, it can be used for dynamic sensing applications where continuous monitoring is required. Finally, when employed for obtaining strain frequency response functions, the FPC allows one to achieve higher SNR levels than other methods. More information on the performance of the FPC algorithm is contained in Lamberti *et al.* [43,44,55].

### 2.3. Strain-Based Modal Analysis

A fundamental aspect related to the employment of FBG sensors to dynamic tests is represented by the application of modal analysis techniques capable of relating the measured FBG responses to the dynamic features of the structure under investigation. Since FBGs measure strains, a strain modal formulation has to be adopted [56,57]. According to modal theory, the displacement of a set of physical coordinates can be approximated by the superposition of *m* number of modes:

(6) $$\left\{u\left(t\right)\right\}=\sum_{r=1}^{m}\left\{\phi_{r}\right\}q_{r}\left(t\right)=\left[\Phi \right]\left\{q\left(t\right)\right\}$$

In Equation (6), {u(t)} is the displacement response vector, $\left\{\phi_{r}\right\}$ is the *r*-th modal displacement vector, $q_{r}$ is the the *r*-th generalized modal coordinate, *t* is the time and [Φ] is the displacement modal strain matrix. According to the classical theory of linear elasticity (small displacements), the strain field {ϵ} can be expressed in terms of the displacement {u} by means of the following relation:

(7) $$\left\{\epsilon \right\}=S\left\{u\right\}=\frac{1}{2}\left(\nabla +\nabla^{T}\right)\left\{u\right\}$$

where ∇ is the linear differential operator in the spatial domain and the superscript *T* indicates the transpose. A similar relation exists between the modal displacement vector $\left\{\phi_{r}\right\}$ and the modal strain vector $\left\{\psi_{r}^{\epsilon }\right\}$:

(8) $$\left\{\psi_{r}^{\epsilon }\right\}=S\left\{\phi_{r}\right\}$$

Using Equations (7) and (8), Equation (6) can be rewritten as:

(9) $$\left\{\epsilon \left(t\right)\right\}=\sum_{r=1}^{m}\left\{\psi_{r}^{\epsilon }\right\}q_{r}\left(t\right)=\left[\Psi^{\epsilon }\right]\left\{q\left(t\right)\right\}$$

where $\left[\Psi^{\epsilon }\right]$ is the modal strain matrix.

Moreover, the relationship between $q_{r}$ and the load vector {F(t)} is:

(10) $$q_{r}\left(t\right)={\left(-\omega^{2}m_{r}+k_{r}+2j\omega \xi_{r}\sqrt{k_{r}m_{r}}\right)}^{-1}{\left\{\phi_{r}\right\}}^{T}\left\{F\right\}=\gamma_{r}{\left\{\phi_{r}\right\}}^{T}\left\{F\left(t\right)\right\}$$

where *ω* is the excitation angular frequency, $m_{r}$, $k_{r}$ and $\xi_{r}$ are, respectively, the *r*-th modal mass, modal stiffness and modal damping ratio, and $\gamma_{r}={\left(-\omega^{2}m_{r}+k_{r}+2j\omega \xi_{r}\sqrt{k_{r}m_{r}}\right)}^{-1}$. The physical meaning of $\gamma_{r}$ is the frequency response function of the same system if $q_{r}$ is considered as the response of an equivalent single degree of freedom system. Substituting Equation (10) back into Equation (9) and dividing by the load {F(t)} yields the definition of the strain frequency response function (SFRF) matrix:

(11) $$\left[H^{\epsilon }\right]=\left[\Psi^{\epsilon }\right]\left[\Gamma \right]{\left[\Phi \right]}^{T}=\sum_{r=1}^{m}\gamma_{r}\left\{{\psi^{\epsilon }}_{r}\right\}{\left\{\phi_{r}\right\}}^{T}$$

Equation (11) can be expanded as:

(12) $$\left[\begin{array}{cccc} H_{11}^{\epsilon } & H_{12}^{\epsilon } & \cdots & H_{1N_{i}}^{\epsilon }\\ H_{21}^{\epsilon } & H_{22}^{\epsilon } & \cdots & H_{2N_{i}}^{\epsilon }\\ \vdots & \vdots & \vdots & \vdots \\ H_{N_{o}1}^{\epsilon } & H_{N_{o}2}^{\epsilon } & \cdots & H_{N_{o}N_{i}}^{\epsilon } \end{array}\right]=\sum_{r=1}^{m}\;\left[\begin{array}{cccc} \psi_{1r}^{\epsilon }\phi_{1r} & \psi_{1r}^{\epsilon }\phi_{2r} & \cdots & \psi_{1r}^{\epsilon }\phi_{N_{i}r}\\ \psi_{2r}^{\epsilon }\phi_{1r} & \psi_{2r}^{\epsilon }\phi_{2r} & \cdots & \psi_{2r}^{\epsilon }\phi_{N_{i}r}\\ \vdots & \vdots & \vdots & \vdots \\ \psi_{N_{o}r}^{\epsilon }\phi_{1r} & \psi_{N_{o}r}^{\epsilon }\phi_{N_{o}r} & \cdots & \psi_{N_{o}r}^{\epsilon }\phi_{N_{i}r} \end{array}\right]$$

where $N_{i}$ indicates the number of excitation points (input points) and $N_{o}$ represents the number of points where the strain is measured (output points). The physical meaning of the term $H_{ij}^{\epsilon }$ is the strain response induced at point *i* by a unit load acting at point *j*. In general, the SFRF matrix $H^{\epsilon }$ is not a square matrix, and differently from the FRF matrix, it is not symmetric, *i.e.*, $H_{ij}^{\epsilon }\neq H_{ji}^{\epsilon }$. This means that reciprocity is not guaranteed. Any row of $H^{\epsilon }$ contains all of the information regarding the displacement mode shape $\phi_{r}$, while any column contains all of the information about the strain mode shape $\psi_{r}$. This particular propriety means that the strain mode shape can be obtained by using one optical fiber line with several spatially-multiplexed FBGs and one fixed excitation point. Often, FBG sensors are employed to measure strain in operational conditions. In this situation, the input forces are unknown, and the output measured strain is the only information available. In this case, the deterministic knowledge of the input is replaced by the assumption that the input is characterized by a constant power spectrum independent of the frequency *ω*. Under this assumption, it is possible to demonstrate that the power spectrum $S_{\epsilon \epsilon }$ of the output is proportional to the square of the SFRF [58]:

(13) $$S_{\epsilon \epsilon }\left(\omega \right)=H^{\epsilon }\left(\omega \right)S_{FF}{H^{\epsilon }}^{H}\left(\omega \right)$$

where $S_{FF}$ is the the input power spectrum and the superscript H indicates the Hermitian operator. When Equation (13) holds, the output power spectrum $S_{\epsilon \epsilon }$ can be used for modal parameter estimation. Due to the similarities between classic and strain modal analysis, which were described above, the same modal parameter estimators can be used. In this article, the Peak-Picking [45] and PolyMax [46,47] modal parameter estimators are used. In the Peak-Picking technique, the modal frequencies are identified from the positions of the peaks in the magnitude of the SFRF, while the damping ratios are computed from the half power (−3dB) points of the SFRF magnitude. This method is very fast and straightforward, but its accuracy is dependent on the frequency resolution used for the measurement. Moreover, it does not provide optimal results when the modal separation is too low or when the measurements are affected by leakage. On the other hand, the PolyMax estimator is much more advanced. Based on a poly-reference least-squares complex frequency estimation, it overcomes the limitations of the Peak-Picking method and allows highly accurate modal parameter estimation, even when the frequency resolution is poor and the modal density is high. One of the key advantages of the PolyMax estimator is that it provides very clean stabilization diagrams. These diagrams are useful tools to separate the modal parameters associated with physical vibration modes from those caused by mathematical artifacts. A stabilization diagram is constructed by repeating the modal analysis for increasing the model order (the number of modes *m* considered for the analysis) and by retaining only the system parameters (also known as system poles) that are stable (*i.e.*, with a negative real part) between one estimation and another. Mathematical poles induced by noise in the measurements appear to jump around in the stabilization diagram without being stable for all of the model orders. The PolyMax method used in this article has the interesting property that the non-physical poles are estimated with a negative damping ratio, so that they can be automatically excluded. An exhaustive explanation of the PolyMax estimator is outside the scope of this paper. Additional information can be found in [46,47].

## 3. Manufacturing of the Composite Components

### 3.1. Carbon Fiber-Reinforced Automotive Control Arm

**Figure 3:**
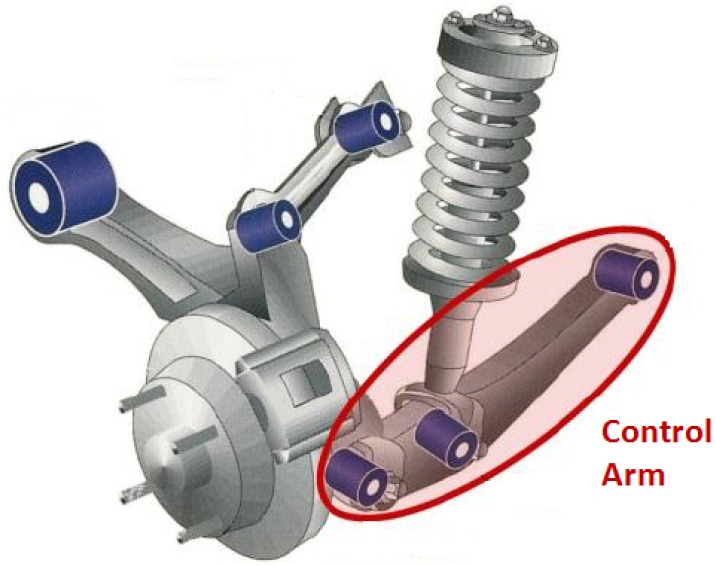
Schematic of the car control arm (source Concept Motors ltd., Essex, UK).

The CFRP component used in this work is an automotive control arm part of a rear wheel suspension system (Figure 3).

**Figure 4:**
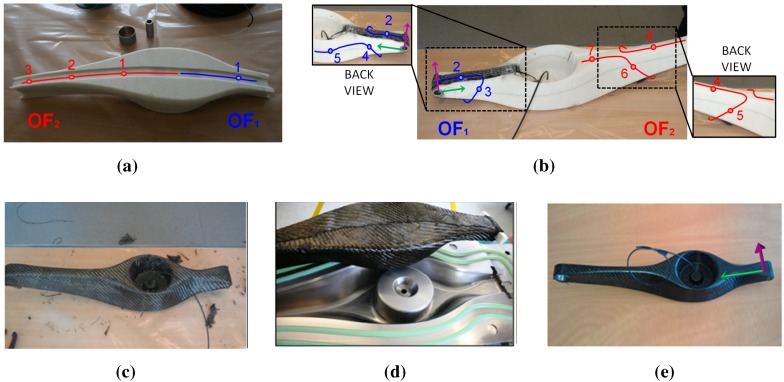
Overview of the CFRP automotive control arm production by the resin transfer molding (RTM) technique. PET core bottom (**a**) and top/lateral (**b**) sides with OF${}_{1}$ (OF, optical fiber) (blue) and OF${}_{2}$ (red); preform before resin injection (**c**); preform insertion in the mold (**d**); final results after demolding (**e**). The arrows in (**a**,**e**) indicate the longitudinal (green) and transverse (magenta) axes.

The design of this CFRP control arm was carried out within the framework of the European research project Cornet DeMaCo [59]. The component is made up of a PET core (Figure 4a,b) reinforced with external carbon/epoxy skins and containing two metallic bushings inserted at its extremities.

The production of the CFRP component started with the preparation of the foam core by CNCmilling. Successively, the two metallic insertions were put in place using two unidirectional (UD) layers of dry carbon fibers (Figure 4b) running along the whole foam perimeter. After this, a carbon fiber twill layer was draped over the entire core surface, and two optical fibers (OF${}_{1}$ and OF${}_{2}$) with a diameter of 125 µm were embedded. OF${}_{1}$ (blue line in Figure 4a,b) contained five FBGs, while OF${}_{2}$ (red line in Figure 4a,b) carried seven FBGs. The gratings were equally spaced with 9 cm in between two consecutive gratings. Table 1 reports the location of each FBG sensor on the CFRP control arm and its position in the lay-out. During the sensor placement, five FBGs (FBG${}_{1}$ and FBG${}_{2}$ of OF${}_{1}$; FBG${}_{1}$, FBG${}_{2}$ and FBG${}_{4}$ of OF${}_{2}$) were embedded along the component longitudinal direction in order to achieve higher sensitivity to bending loads (acting on the longitudinal axis). At the same time, four FBGs (FBG${}_{3}$, FBG${}_{4}$ of OF${}_{1}$; FBG${}_{5}$ and FBG${}_{6}$ of OF${}_{2}$) were embedded with an orientation of 45${}^{\circ }$ in order to enhance their sensitivity to torsion loads (acting around the longitudinal axis). After the embedding of the optical sensors, the preformed CFR (Figure 4c) was inserted inside a metallic mold (Figure 4d) where a preheated resin at 40 °C was injected at a pressure of 0.5 MPa. After curing, the component was demolded and post-cured in an oven for 48 h at 50 °C. Once the production was terminated, the integrity of the optical fibers was verified.

**Table 1:** Location of the FBGs on the control arm and their position in the lay-out. The 5 FBGs embedded in the longitudinal direction are more sensitive to bending, while the 4 FBGs embedded under 45${}^{\circ }$ are more sensitive to torsion. FBG${}_{3}$ of OF${}_{2}$ is located over the bushing region. The 2 extra FBGs are placed according to the fibers egress points.

Fiber Line	Grating	Location on the Arm	Position in the Lay-Out
OF${}_{1}$	FBG${}_{1}$	longitudinal bottom	2nd UD/3rd UD
	FBG${}_{2}$	longitudinal top	2nd UD/3rd UD
	FBG${}_{3}$	45${}^{\circ }$ side	foam/twill
	FBG${}_{4}$	45${}^{\circ }$ side	foam/twill
	FBG${}_{5}$	extra FBG	foam/twill
OF${}_{2}$	FBG${}_{1}$	longitudinal bottom	2nd UD/3rd UD
	FBG${}_{2}$	longitudinal bottom	2nd UD/3rd UD
	FBG${}_{3}$	side	2nd UD/3rd UD
	FBG${}_{4}$	longitudinal top	2nd UD/3rd UD
	FBG${}_{5}$	45${}^{\circ }$ side	foam/twill
	FBG${}_{6}$	45${}^{\circ }$ side	foam/twill
	FBG${}_{7}$	extra FBG	foam/twill

**Figure 5:**
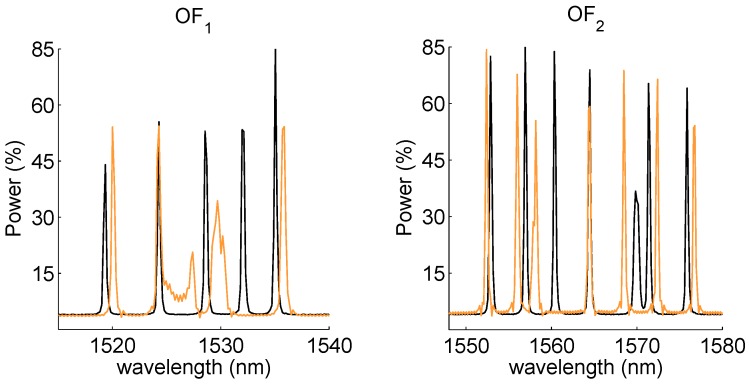
Reflected spectra for OF${}_{1}$ (left) and OF${}_{2}$ (right), before embedding (black) and after production (orange). The third and forth FBGs of OF${}_{1}$ are subjected to peak broadening and distortion induced by non-uniform thermomechanical loads generated during the production process.

Figure 5 shows the reflected spectra of the two OFs before the embedding in the control arm and after production (*i.e.*, after post-curing). The spectra were acquired with an FBGS scan FBG804D interrogator [42], which has a wavelength range of 1510–1590 nm and a wavelength resolution of 156 pm. Because of thermal gradients and non-uniform stresses generated during the production process (both curing and post-curing), distortions occurred for two of the FBGs of OF${}_{1}$, FBG${}_{3}$ and FBG${}_{4}$. This is mainly due to the resin curing. In fact, FBG${}_{3}$ and FBG${}_{4}$ are embedded in direct contact with the PET foam where the resin uptake is expected to be higher. In particular, the significant power reduction of FBG${}_{3}$ is indicative of a non-uniform stress distribution acting along the FBG${}_{3}$ grating length, while the peak-splitting (*i.e.*, induced birefringence) of FBG${}_{4}$ denotes the presence of transverse stresses (*i.e.*, pressure) setting in because of the resin curing. The FBGs embedded between two UD layers reveal a lower shrinkage thanks to the supporting stiffness provided by the carbon fibers. Table 2 reports the Bragg wavelengths for all of the embedded FBG sensors obtained in the initial and final (post production) condition.

**Table 2:** FBG center wavelengths during the CFRP component production.

Fiber Line	Grating	Initial $\lambda_{B}$ (nm)	Final $\lambda_{B}$ (nm)
OF${}_{1}$	FBG${}_{1}$	1519.313	1520.011
	FBG${}_{2}$	1524.290	1524.251
	FBG${}_{3}$	1528.621	1527.412
	FBG${}_{4}$	1532.059	1529.695
	FBG${}_{5}$	1535.020	1535.854
OF${}_{2}$	FBG${}_{1}$	1552.906	1552.393
	FBG${}_{2}$	1556.958	1555.989
	FBG${}_{3}$	1560.377	1558.166
	FBG${}_{4}$	1564.455	1564.492
	FBG${}_{5}$	1569.884	1568.468
	FBG${}_{6}$	1571.374	1572.412
	FBG${}_{7}$	1575.847	1576.779

### 3.2. Glass Fiber Reinforced Aeronautic Hinge Arm

The second component analyzed in this research is a GFRP hinge arm, which is part of a wing-slat system. Such a component was designed in collaboration with the company ASCO [60] and manufactured via the same RTM technique used for the CFRP component.

**Figure 6:**
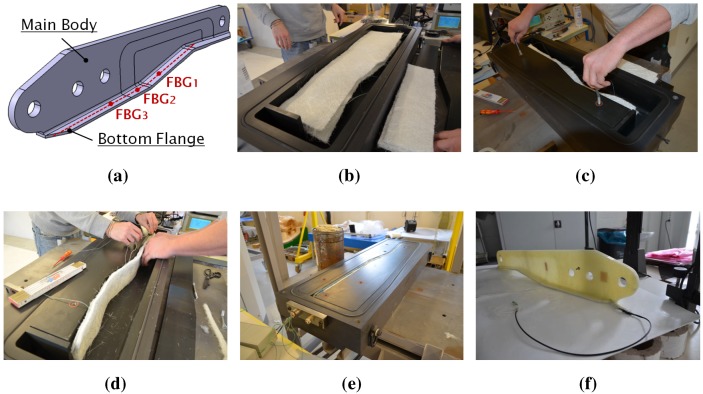
Design and production of the GFRP aeronautic hinge arm. The hinge arm CAD model with embedded optical fiber sensors (red line) in the bottom flange (**a**); main body insertion (**b**) and fixation (**c**) inside the metallic mold; embedding of the FBG sensors inside the bottom flange (**d**); closing of the mold (**e**); demolding and final result (**f**).

**Figure 7:**
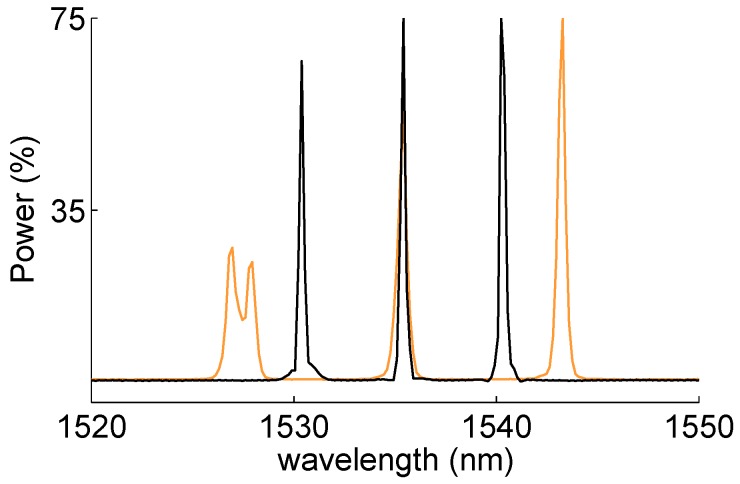
FBG reflected spectra before embedding (black) and after production (orange) for the GFRP component. FBG${}_{1}$ is affected by peak splitting and broadening induced by non-uniform stresses generated during the manufacturing process.

It must be noted that in the original design, this hinge arm is made of carbon fiber reinforcements. However, to provide a proof of concept and to contain the cost at the same time, this research was conducted on a preliminary prototype made of GFRP. Figure 6 shows a CAD picture of the GFRP component and the different steps of its production process. Glass fiber reinforcements were initially placed inside a metallic mold. At the same time, one optical fiber (diameter 125 µm) with three equally-spaced (10 cm) FBG sensors was embedded along the center-line of the component bottom flange mid-plane (Figure 6d). Given the flexural rigidity of the GFRP component, the FBG sensors were multiplexed along the component longitudinal direction, in order to make them more sensitive to the component main longitudinal bending modes. After the sensor placement, the mold was closed, and an epoxy resin from Huntsman Araldide LY1564 was injected at 0.5 MPa. Before the injection, the resin was preheated at 60 °C. The hinge arm was cured for 24 h at room temperature and post-cured for 8 h at 80 °C. Figure 7 shows the FBG spectra acquired using an FBGS scan FBG804D before embedding and after demolding. The Bragg wavelengths in initial (before embedding) and final (after demolding) conditions are reported in Table 3. The reflected peak of the first grating (FBG${}_{1}$) exhibits evident birefringent effects together with peak broadening and distortion caused by the internal non-uniform stresses generated during the component manufacturing. FBG${}_{1}$ can still be used for the analysis. In particular, the two peaks FBG${}_{1}$ and FBG${}_{1^{*}}$ should move together, since they are associated with the same physical grating; therefore, they should produce very similar results. As will be shown in Section 4.3, this statement is verified when the FPC algorithm is used, but not when the MD demodulation is employed. This is mainly due to the intrinsic lower precision of the MD algorithm.

**Table 3:** FBG center wavelengths during the GFRP component production. Birefringence occurs for FBG${}_{1}$. The asterisk ∗ indicates the second peak associated with FBG${}_{1}$.

Grating	Initial $\lambda_{B}$ (nm)	Final $\lambda_{B}$ (nm)
FBG${}_{1}$	1530.389	1526.837
		1527.776 *
FBG${}_{2}$	1535.413	1535.235
FBG${}_{3}$	1540.398	1542.806

## 4. Experiments and Results

### 4.1. Experimental Setup and Procedure

**Figure 8:**
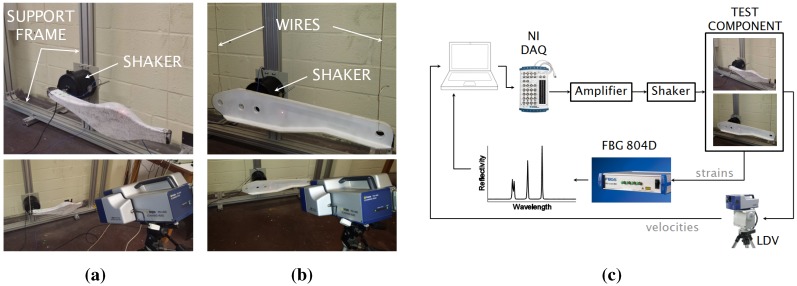
Experimental setup for the control arm (**a**) and the hinge arm (**b**); experimental procedure (**c**) adopted for both composite components.

The CFRP control arm and the GFRP hinge arm were tested to retrieve their modal parameters (modal frequencies and damping ratios). The modal parameter estimation was performed using the strain measured by the embedded FBGs together with the out of plane velocities acquired via a laser Doppler vibrometer (LDV). In particular, the Polytec PSV-400 laser Doppler vibrometer was used. The experimental setup and the procedure adopted to test both components are displayed in Figure 8.

Each component was suspended to a support aluminum frame by means of two flexible wires. To increase the quality of the LDV measurements, the surface reflectivity of the two components was improved by means of ARDROX^®^ reflective spray, which consists of a suspension of an inert white powder in a quick-drying solvent. An electromechanical shaker was mounted on the support frame and attached to the component under test (first to the CFRP and successively to the GFRP arm). A Schroeder multisine [61] up to 500 Hz was generated in MATLAB^®^, amplified and sent to the shaker through an NI USB-6341 [62] data acquisition card. During the excitation, the LDV measured the out of plane velocities at a rate of 4 kHz. At the same time, the FBG 804D interrogator acquired and stored the FBGs’ reflected spectra with a sampling frequency of 1 kHz, using an in-house-developed MATLAB code. The acquired spectra were successively demodulated by the MD and FPC algorithms described in Section 2.2. To compute the strain time histories associated with each FBG sensor, appropriate wavelength windows were applied to the acquired reflected spectra. Wavelength windows with a 2 nm bandwidth and centered around each FBG initial Bragg wavelength were used for all FBG sensors. The strain and velocity power spectra were eventually computed and analyzed by both the Peak-Picking and PolyMax estimators. It is worth noticing that the adopted Shroeder multisine excitation has a flat power spectrum over the frequency *ω*; therefore, Equation (13) holds. For this reason, the PolyMax estimator was directly applied to the measured output signals (strains from FBGs and velocities from LDV).

Another observation that has to be done concerns the decision of performing operational modal analysis (using output-only measurements), rather than experimental modal analysis (measuring also the excitation). In effect, the main issue of performing experimental modal analysis consists of achieving a perfect synchronization of FBG, LDV and force transducer measurements. The authors are updating the MATLAB script used in this research in order to allow the acquisition of synchronized data for future works. Nevertheless, the output-only measurements performed in this article are a valid demonstration of the feasibility of using FBG sensors for vibration measurements in operating conditions. The procedure described above was used first for the CFRP component and successively for the GFRP aeronautic hinge arm.

### 4.2. Modal Analysis of the CFRP Automotive Component

**Figure 9:**
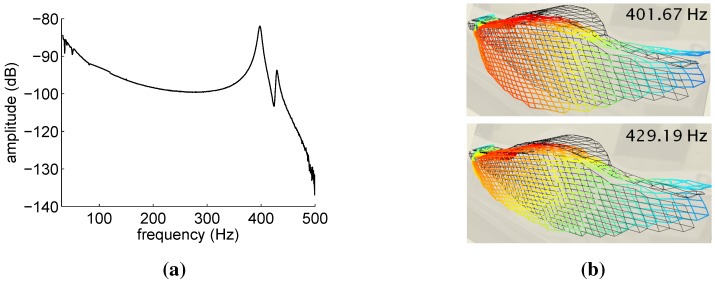
Reference results for the automotive control arm obtained via laser Doppler vibrometer (LDV) velocity measurements. Frequency response magnitude obtained by averaging all of the scanned points (**a**) and vibration mode shapes (**b**).

The CFRP automotive control arm was tested using the experimental procedure described in Section 4.1, during which the reflected spectra of the 12 embedded FBG sensors and the out of plane surface velocities were measured and stored. Figure 9 shows the reference results obtained by means of the LDV velocities measurements. The magnitude of the LDV frequency response (Figure 9a) clearly indicates the presence of two resonances in the analyzed frequency bandwidth 0–500 Hz. The vibration mode shapes corresponding to each of these resonances were obtained using the geometry scanning head of the PSV-400 and are reported in Figure 9b. A pure longitudinal bending mode is associated with the first resonance, while a coupled bending-torsional mode corresponds to the second resonance.

Table 4 compares the component modal parameters (frequencies and damping ratios) computed from the LDV measurements using both Peak-Picking and Polymax estimation techniques. Due to the LDV frequency resolution (0.250 Hz) and sensitivity, the performance of the Peak-Picking method is comparable to that of the PolyMax estimator.

**Table 4:** Modal parameters of the automotive composite component estimated from LDV measurements. Comparison between the Peak-Picking and PolyMax estimators.

Frequency *f* (Hz)		Damping Ratio *ξ* (%)	
Peak-Picking	PolyMax	Peak-Picking	PolyMax
398.250	398.791	0.604	0.976
428.750	429.194	0.575	0.709

**Figure 10:**
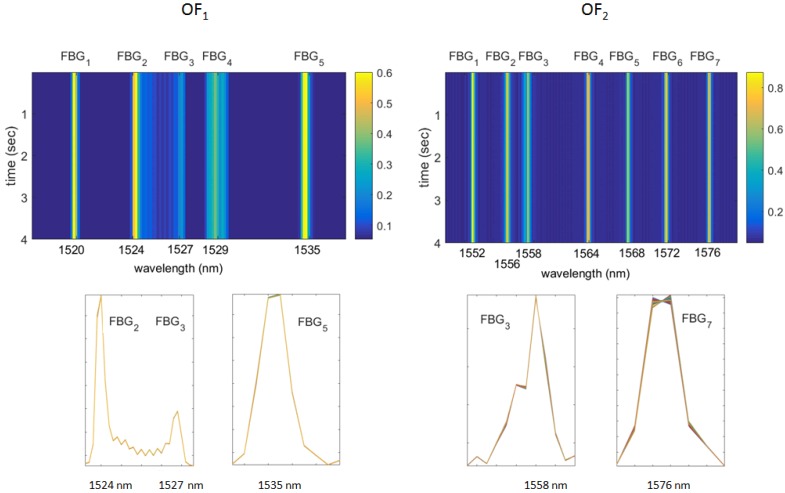
Reflected spectra of OF${}_{1}$ (left column) and OF${}_{2}$ (right column) during four seconds of excitation. Reflectivity maps *vs*. time and wavelength (top row); reflected signals modifications for three gratings of OF${}_{1}$ and for two gratings of OF${}_{2}$ (bottom row) caused by the applied load.

Figure 10 shows the FBG spectra measured and stored during the multisine excitation. It can be observed that the shape of the FBG reflected spectra remains almost identical during the loading of the component, meaning that the strain acting on the different gratings is approximately uniform. The variations of the reflected spectra are more pronounced for OF${}_{2}$ (due to higher strain levels) rather than for OF${}_{1}$ (Figure 10 bottom row); therefore, OF${}_{2}$ is expected to produce better results than OF${}_{1}$.

**Figure 11:**
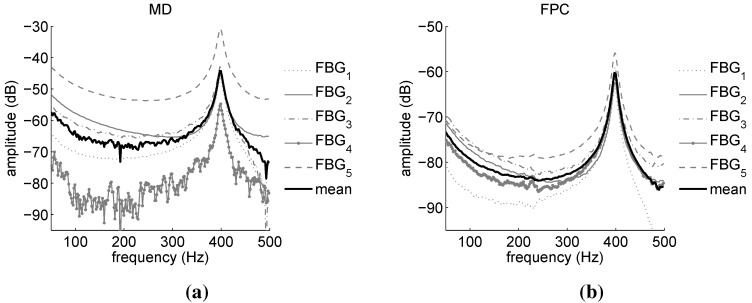
Automotive component: power spectra amplitudes obtained via the FBG sensors of OF${}_{1}$. Comparison between maximum-detection (MD) (**a**) and FPC (**b**) performance.

**Figure 12:**
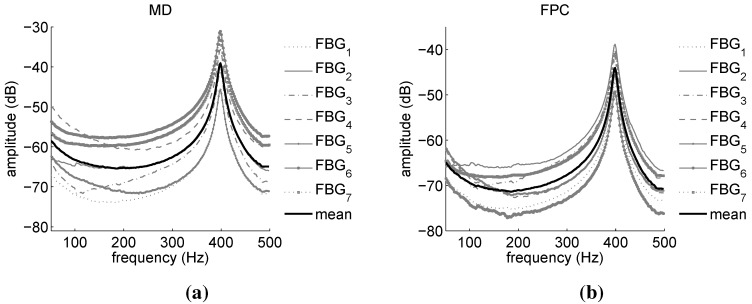
Automotive component: power spectra amplitudes obtained via the FBG sensors of OF${}_{2}$. Comparison between MD (**a**) and FPC (**b**) performance.

Figure 11 and Figure 12 report the power spectra calculated from the demodulation of the FBG signals of Figure 10 by means of the MD and FPC algorithms. Figure 11 refers to the FBGs of OF${}_{1}$, while Figure 12 is associated with the FBGs of OF${}_{2}$.

For OF${}_{1}$, the FPC performs much better than the MD, especially in the case of FBG${}_{4}$. In this instance, in fact, the output of the MD algorithm is deteriorated by the distortion affecting the FBG${}_{4}$ reflected peak (see Figure 5), resulting therefore in being noisy. On the contrary, the SNR of the FPC output is still considerably higher. In the case of OF${}_{2}$, the MD and FPC performances are comparable, since the reflected spectra of the OF${}_{2}$ gratings are sharp and undistorted (see Figure 5). The mean value of the modal parameters identified on the basis of the FBG power spectra are summarized in Table 5. Peak-Picking and PolyMax estimates are reported for both MD and FPC. The pick-picking values reported in Table 5 have been obtained by first processing the FBGs responses one by one and by successively computing the mean values. For the PolyMax estimates, instead, all of the FBG responses have been simultaneously considered and one global estimate retrieved.

**Table 5:** Modal parameters of the automotive composite component estimated from FBG measurements using both the MD and FPC algorithms. Comparison between the Peak-Picking and PolyMax estimators.

Frequency *f* (Hz)				Damping Ratio *ξ* (%)			
MD Measurements							
Peak-Picking		PolyMax		Peak-Picking		PolyMax	
OF${}_{1}$	OF${}_{2}$	OF${}_{1}$	OF${}_{2}$	OF${}_{1}$	OF${}_{2}$	OF${}_{1}$	OF${}_{2}$
398	399	398.366	398.473	1.080	1.203	0.886	0.883
FPC Measurements							
Peak-Picking		PolyMax		Peak-Picking		PolyMax	
OF${}_{1}$	OF${}_{2}$	OF${}_{1}$	OF${}_{2}$	OF${}_{1}$	OF${}_{2}$	OF${}_{1}$	OF${}_{2}$
398	398	398.363	398.475	1.206	1.203	0.896	0.885
-	-	427.601	429.852	-	-	0.335	0.685

The second natural frequency and damping ratio cannot be identified using the Peak-Picking technique, since the FBG power spectra show only one clearly visible resonance. However, the second resonance can be identified when the PolyMax estimator processes the FBG data demodulated with the FPC algorithm. Therefore, the combination FPC-PolyMax guarantees the best results. The fact that the FBG spectra of Figure 11 and Figure 12 do not clearly show the second resonance captured by the LDV is due to the torsion strains induced by the applied load, which are close to the FBG strain sensitivity limit. On the contrary, the LDV high sensitivity allows one to detect the surface velocities associated with torsional vibrations. Table 6 reports the accuracy of the FBG-PolyMax measurements with respect to the LDV-PolyMax results of Table 4 (the accuracy of the Peak-Picking method is not reported due to its evident worst estimation performance). Since the accuracy is defined as percentage of variation, lower values in Table 6 indicate better accuracy. The modal frequencies are estimated much more accurately than the damping ratios. The accuracy on the frequencies is always better than 0.4%. The worst accuracy regards the estimation of the damping ratio $\xi_{2}$ obtained via the OF${}_{1}$.

**Table 6:** Automotive component: accuracy of the FBG-PolyMax outputs with respect to LDV-PolyMax reference results. Comparison between MD and FPC performance.

$|f_{LDV}-f_{FBG}|/f_{LDV}$ (%)		$|\xi_{LDV}-\xi_{FBG}|/\xi_{LDV}$ (%)	
MD-PolyMax			
OF${}_{1}$	OF${}_{2}$	OF${}_{1}$	OF${}_{2}$
0.106	0.079	9.22	9.52
FPC-PolyMax			
OF${}_{1}$	OF${}_{2}$	OF${}_{1}$	OF${}_{2}$
0.107	0.079	8.196	9.32
0.371	0.153	52.75	3.385

### 4.3. Modal Analysis of the GFRP Aeronautic Component

**Figure 13:**
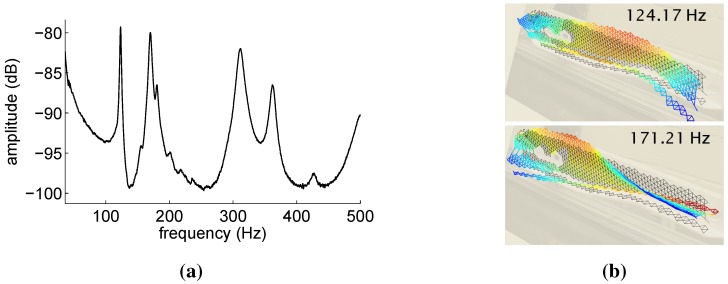
Reference results for the aeronautic hinge arm obtained via LDV velocity measurements. Frequency response magnitude obtained by averaging all of the scanned points (**a**) and vibration mode shapes (**b**).

The aeronautic hinge arm was tested using the same experimental procedure adopted for the automotive component and outlined in Section 4.1. Figure 13 reports the reference results obtained by means of the LDV velocities measurements. The magnitude of the LDV frequency response (Figure 13a) shows several resonances in the analyzed frequency bandwidth 0–500 Hz. The vibration mode shapes associated with the first two of these resonances are reported in Figure 13b. The first mode is a a bending mode, while the second is torsional. Table 7 compares the component modal parameters (frequencies and damping ratios) computed from the LDV measurements using both Peak-Picking and Polymax estimation techniques. As for the automotive component case, due to the LDV frequency resolution (0.250 Hz) and sensitivity, the performances of the two estimation techniques are quite similar, except for the case of $\xi_{2}$, whose value could not be determined with the Peak-Picking method. It is worth noticing that the values reported in Table 7 are the first five stable system modal parameters contained in the analyzed frequency bandwidth. The PolyMax estimator automatically provides only these values by excluding all of the non-physical system poles, such as those contained in the bandwidth 200–300 Hz. On the contrary, in the case of the Peak-Picking method, such non-physical poles have to be manually excluded by the operator. The correctness of the poles automatically estimated with the PoliMax method is supported by the fact that to each of these poles corresponds a physical vibration mode shape (the first two mode shapes are shown in Figure 13b).

**Table 7:** First five modal parameters of the aeronautic composite component estimated from LDV measurements. Comparison between Peak-Picking and PolyMax estimators.

Frequency *f* (Hz)		Damping Ratio *ξ* (%)	
Peak-Picking	PolyMax	Peak-Picking	PolyMax
123.038	124.084	0.863	0.817
170.053	171.362	-	1.377
180.556	181.554	0.843	0.871
311.347	311.864	1.804	1.563
362.113	363.983	1.437	1.144

**Figure 14:**
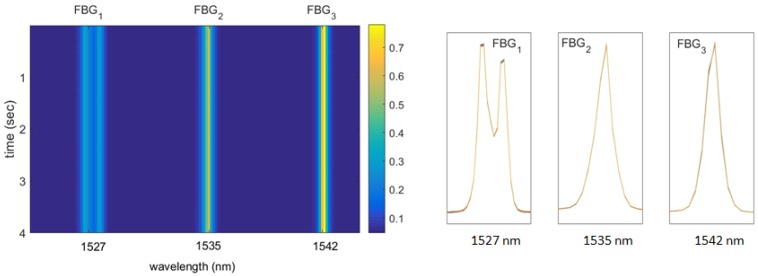
Reflected spectra during four seconds of excitation. Reflectivity map *vs*. time and wavelength (left); reflected signals modifications (right) caused by the applied load.

Figure 14 shows the FBG spectra measured and stored during the multisine excitation. As for the case of the automotive control arm, the shape of the FBG reflected spectra remains almost identical during the excitation, which indicates approximately uniform strain acting on the gratings.

**Figure 15:**
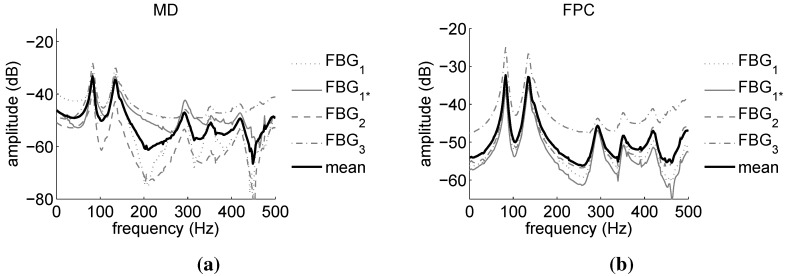
Aeronautic component: power spectra amplitudes obtained via the FBG sensors. Comparison between MD (**a**) and FPC (**b**) performance. FBG${}_{1}$ and FBG${}_{1^{*}}$ power spectra should be quite similar, since they are associated with the same grating. This does not happen in the case of the MD due to the poor precision of the algorithm.

Figure 15 displays the power spectra calculated from the demodulation of the FBG signals of Figure 14 by means of the MD (Figure 15a) and FPC (Figure 15b) algorithms.

Both power spectra are very similar to the LDV magnitude of Figure 13a, although in them, the third resonance is barely visible. This resonance is therefore impossible to identify using the Peak-Picking estimation method. This is confirmed by the results reported in Table 8, where the mean value of the component first five modal parameters identified on the basis of the FBG power spectra are summarized. Peak-Picking and PolyMax estimates are reported for both MD and FPC. Differently from the Peak-Picking, the PolyMax estimator not only successfully identifies all of the modal parameters, but it is also more accurate. In fact, the Peak-Picking accuracy is limited by the measurement frequency resolution (1 Hz). On the contrary, the PolyMax estimator allows one to achieve sub-Hz accuracy. Table 9 reports the accuracy of the FBG-PolyMax measurements with respect to the LDV-PolyMax results of Table 7 (the accuracy of the Peak-Picking method is not reported due to its evident worst estimation performance). As in the case of the automotive component, the estimation of the modal frequencies is considerably more accurate than the damping ratios’ identification. The combination FPC-PolyMax produces more accurate estimates than the combination MD-PolyMax in four cases out of five for the modal frequencies and in three cases out of five for the damping ratios.

**Table 8:** First five modal parameters of the aeronautic composite component estimated from FBG measurements using both the MD and FPC algorithms. Comparison between the Peak-Picking and PolyMax estimators. Peak-Picking is not able to estimate either $f_{3}$ or  $\xi_{3}$.

Frequency *f* (Hz)		Damping Ratio *ξ* (%)	
MD Measurements			
Peak-Picking	PolyMax	Peak-Picking	PolyMax
124	124.159	2.016	0.867
170	171.325	2.205	1.422
-	180.278	-	1.416
312	311.398	1.442	1.541
365	362.812	2.937	1.026
FPC Measurements			
Peak-Picking	PolyMax	Peak-Picking	PolyMax
124	124.136	2.016	0.885
170	170.972	2.205	1.401
-	180.442	-	1.357
312	311.767	1.923	1.601
364	363.245	3.142	1.104

**Table 9:** Aeronautic component: accuracy of the FBG-PolyMax outputs with respect to LDV-PolyMax reference results. Comparison between MD and FPC performance.

$|f_{LDV}-f_{FBG}|/f_{LDV}$ (%)		$|\xi_{LDV}-\xi_{FBG}|/\xi_{LDV}$ (%)	
MD-PolyMax			
0.060		6.120	
0.138		6.357	
0.703		62.57	
0.786		1.407	
0.322		10.31	
FPC-PolyMax			
0.042		8.323	
0.344		4.786	
0.612		55.79	
0.668		2.431	
0.203		3.496	

## 5. Conclusions

In this work, the capability of embedded fiber Bragg grating (FBG) sensors to perform the modal analysis of real-life industrial composite components was investigated. The first component under test was a CFRP control arm used for an automotive rear wheel suspension system. The second component considered for the analysis was a GFRP hinge arm, designed to be incorporated inside a wing leading-edge high lift device. Both components were manufactured via resin transfer molding (RTM) and instrumented with embedded multiplexed FBG sensors. The modal analysis was performed by exciting the two components with a shaker and by processing the FBG dynamical measurements with state-of-the-art algorithms. For the demodulation of the FBG signals, two different algorithms were employed: the first is a maximum detection (MD) algorithm, which is conventionally implemented in several FBGs interrogators; the second is the fast phase correlation (FPC) algorithm recently developed by the authors. For the modal analysis, two estimation techniques were used: Peak-Picking and PolyMax. The analysis of the results showed that:

- The FPC demodulation performed better than the MD, especially in the case of a distorted reflected Bragg peak. Particularly, the FPC provided a signals with better signal-to-noise ratios than those obtained via the MD, making, therefore, the identification of the modal parameter more accurate.
- The PolyMax estimator processed the FBG demodulated signals and identified the component modal parameters better than the Peak-Picking estimator. Peak-Picking was not able to retrieve either the second resonance of the automotive control arm or the third resonance of the aeronautic hinge arm. This resonance could be identified with the PolyMax technique.
- The combination FPC-PolyMax guaranteed the most accurate results, being able to treat both distorted and undistorted FBG peaks and to identify modal parameters associated even with barely visible structural resonance. The MD-PolyMax resulted in being less accurate and even failed in one instance.
- The estimation of the component modal frequencies was in general one order of magnitude more accurate than the identification of the damping ratios. This is a well-established phenomenon in modal analysis.

All of these results confirm that fiber Bragg grating sensors can be efficiently used for the dynamic characterization of complex real-life structures. Moreover, with the appropriate selection of processing algorithms, they can be valid substitutes of LDV measurements, especially for those cases where the accessibility to the test structures is an issue. In particular, the selection of the FBG demodulation algorithm is a crucial aspect. A good selection has to take into account the particular type of application the user is faced with. For instance, in the cases of surface-mounted FBG sensors interrogated with a high resolution device and expected to undergo high strain levels, the MD algorithm is still a good choice, since it will produce results similar to those achievable with more advanced algorithms like the FPC. On the other hand, for applications where spectral distortions are expected (FBG embedded in composites or exposed to harsh environments) or where the available interrogation device is limited in wavelength resolution, advanced algorithms like the FPC can really make the difference compared to a conventional MD peak tracking method. Future works related to this research will be focused on the exploitation of the embedded FBGs as *in situ* sensors for damage detection and monitoring purposes. The view is to start an experimental campaign focused on the fatigue testing of the two composite components and on the use of the embedded FBGs to detect and monitor incipient and propagating damage (such as delaminations).

## References

[1] Berthelot J.M.O. (1999). Photosensitivity in Optical Fiber Waveguides: Application to Reflection Filter Fabrication.

[2] Gay D., Hoa S.V., Tsai S.W. (2002). Composite Materials: Design and Applications.

[3] Vassilopoulos A.P., Keller T. (2011). Fatigue of Fiber-Reinforced Composites.

[4] JEC Overview of the Worldwide Composite Industry: 2010–2015, 2011 Release. http://www.jeccomposites.com/product/overview-worldwide-composites-industry-2010-2015-2011-release.

[5] Grattan L.S., Meggit B.T. (2013). Optical Fiber Sensor Technology: Applications and Systems.

[6] Mihailov S.J. (2012). Fiber Bragg Grating Sensors for Harsh Environments. Sensors.

[7] De Pauw B., Lamberti A., Vanlanduit S., van Tichelen K., Geernaert T., Berghmans F. (2014). Signal-to-noise ratio evaluation with draw tower fibre Bragg gratings (DTGs) for dynamic strain sensing at elevated temperatures and corrosive environment. Proc. SPIE.

[8] Elsmann T., Lorenz A., Yazd N.S., Habisreuther T., Dellith J., Schwuchow A., Bierlich J., Schuster K., Rothhardt M., Kido L. (2014). High temperature sensing with fiber Bragg gratings in sapphire-derived all-glass optical fibers. Opt. Express.

[9] Murukeshan V.M., Chan P.Y., Ong L.S., Seah L.K. (2000). Cure monitoring of smart composites using fiber Bragg grating based embedded sensors. Sens. Actuators A Phys..

[10] Leng J.S., Asundi A. (2002). Real-time cure monitoring of smart composite materials using extrinsic Fabry-Perot interferometer and fiber Bragg grating sensors. Smart Mater. Struct..

[11] Olivier P., Mulle M., Paris C., Collombet F. (2012). Carbon/polymeric composites autoclave cure monitoring with optical fiber Bragg grating (FBG) sensors. Wiley Encyclopedia of Composites.

[12] Chiesura G., Luyckx G., Voet E., van Paepegem W., Degrieck J., Kaufmann M., Martens T., Lamberti A., Vanlanduit S. Production monitoring of a RTM automotive control arm by means of fibre optic sensors. Proceedings of the Optimess 2015.

[13] Kinet D., Mégret P., Goossen K.W., Qiu L., Heider D., Caucheteur C. (2014). Fiber Bragg grating sensors toward structural health monitoring in composite materials: Challenges and solutions. Sensors.

[14] Othonos A., Kalli K. (1999). Fibre Bragg Gratings: Fundamentals and Applications in Telecommunications and Sensing.

[15] Cusano A., Cutolo A., Albert J. (2011). Fiber Bragg Grating Sensors: Recent Advancements, Industrial Applications and Market Exploitation.

[16] Luyckx G., Voet E., Lammens N., Degrieck J. (2011). Strain Measurements of Composite Laminates with Embedded Fibre Bragg Gratings: Criticism and Opportunities for Research. Sensors.

[17] Okabe Y., Yashiro S., Kosaka T., Takeda N. (2000). Detection of transverse cracks in CFRP composites using embedded fiber Bragg grating sensors. Smart Mater. Struct..

[18] Takeda S., Okabe Y., Takeda N. (2002). Delamination detection in CFRP laminates with embedded small-diameter fiber Bragg grating sensors. Compos. A.

[19] Jones R., Galea S. (2002). Health monitoring of composite repairs and joints using optical fibers. Compos. Struct..

[20] Studer M., Peters K., Botsis J. (2003). Method for determination of crack bridging parameters using long optical fiber Bragg grating sensors. Compos. B.

[21] Ogisu T., Shimanuki M., Kiyoshima S., Okabe Y., Takeda N. (2005). Damage growth detection of composite laminate using embedded FBG sensor/PZT actuator hybrid system. Proc. SPIE.

[22] Sekine H., Fujimoto S., Okabe T., Takeda N., Yokobori T. (2006). Structural health monitoring of cracked aircraft panels repaired with bonded patches using fiber Bragg grating sensors. Appl. Compos. Mater..

[23] Minakuchi S., Okabe Y., Takeda N. (2007). Real-time detection of Debonding between honeycomb core and facesheet using a small-diameter FBG sensor embedded in adhesive layer. J. Sandw. Struct. Mater..

[24] Sorensen L., Botsis J., Gmur T., Cugnoni J. (2007). Delamination detection and characterisation of bridging tractions using long FBG optical sensors. Compos. A Appl. Sci. Manuf..

[25] Silva-Muñoz R.A., Lopez-Anido R.A. (2009). Structural health monitoring of marine composite structural joints using embedded fiber Bragg grating strain sensors. Compos. Struct..

[26] Bernasconi O., Ewins D.J. Application of strain modal testing to real structures. Proceedings of the 7th International Modal Analysis Conference.

[27] Yam L.Y., Leung T.P., Li D.B., Xue K.Z. (1996). Theoretical and experimental study of modal strain analysis. J. Sound Vib..

[28] Schulz W.L., Conte J.P., Udd E., Kunzler M. Structural damage assessment via modal property identification using macro-strain measurements with fiber Bragg gratings as an alternative to accelerometers. Proceedings of the 15th Optical Fiber Sensors Conference Technical Digest.

[29] Calvert S., Conte J.P., Moaveni B., Schulz W.L., de Callafon R. (2003). Full scale testing results of structural damage detection using long gage fiber Bragg gratings and modal analysis. Proc. SPIE.

[30] Cusano A., Capoluongo P., Campopiano S., Ambrosino C., Giordano M., Caponero M., Paolozzi A., Felli F. (2005). Dynamic measurements on a star tracker prototype of AMS using fiber optic sensors. Proc. SPIE.

[31] Baldwin C.S., Balachandran B., Buckley S. Modal Analysis of Vibrating Structure Using a Fiber Bragg Grating System. Proceedings of the 24th IMAC Conference.

[32] De Pauw B., Vanlanduit S., Berghmans F., Geernaert T., Chah K., van Tichelen K. (2013). Benchmarking of deformation and vibration measurement techniques for nuclear fuel pins. Measurements.

[33] Dos Santos F.L.M., Peeters B., Gielen L., Desmet W., Sandoval Góes L.C. (2015). The use of fiber Bragg grating sensors for strain modal analysis. Topics in Modal Analysis.

[34] Dos Santos F.L.M., Peeters B., van der Vorst R., Desmet W., Sandoval Góes L.C. The use of strain and mixed strain/acceleration measurements for modal analysis. Proceedings of the 9th International Conference on Structural Dynamics.

[35] Moretti P., de Pauw B., Lamberti A., Reynders E., Geernaert T., Berghmans F., de Roeck G. Identification of mode shapes from sub-microstrain Fibre—Ptic Bragg Grating data using an improved wavelength detection algorithm. Proceedings of the Optimess.

[36] Cusano A., Capoluongo P., Campopiano S., Giordano M., Caponero M., Paolozzi A. (2006). Experimental modal analysis of an aircraft model wing by embedded fiber Bragg grating sensors. IEEE Sens. J..

[37] Bang H., Shin H. Structural health monitoring of wind turbine blade using embedded fiber Bragg grating sensors. Proceedings of EWEC2010 Conference.

[38] Lamberti A., Chiesura G., de Pauw B., Vanlanduit S. Monitoring of fatigue induced propagating delaminations using embedded fiber Bragg grating sensors and operational modal parameter estimation. Proceedings of the ICCM20 Conference.

[39] Vella T., Chadderdon S., Selfridge R., Schultz S., Webb S., Park C., Peters K., Zikry M. (2010). Full-spectrum interrogation of fiber Bragg gratings at 100 kHz for detection of impact loading. Meas. Sci. Technol..

[40] Webb S., Peters K., Zikry M.A., Chadderdon S., Nikola S., Selfridge R., Schultz S. (2012). Full-spectral interrogation of fiber Bragg grating sensors exposed to steady-state vibration. Exp. Mech..

[41] Potter K. (1997). Resin Transfer Moulding.

[42] FBG Scan 700. http://www.fbgs.com/products/measurement-devices/fbg-scan-700/800/.

[43] Lamberti A., Vanlanduit S., de Pauw B., Berghmans F. (2014). A novel fast phase correlation algorithm for peak wavelength detection of fiber Bragg grating sensors. Opt. Express.

[44] Lamberti A., Vanlanduit S., de Pauw B., Berghmans F. (2014). Peak detection in fiber Bragg grating using a fast phase correlation algorithm. Proc. SPIE.

[45] Avitabile P. (2006). 101 Ways to exctract modal parameters-which is the one for me?. Exp. Tech..

[46] Peeters B., van der Auweraer H., Guillaume P. (2004). The polymax frequency-domain method: A new standard for modal parameter estimation?. Shock Vib..

[47] Peeters B., Lowet G., van der Auweraer H., Leuridan J. (2004). A new procedure for modal parameter estimation. J. Sound Vib..

[48] PSV. http://www.polytec.com/int/products/vibration-sensors/scanning-vibrometers/.

[49] Kashyap R. (1999). Fiber Bragg Gratings.

[50] Barlow A., Payne D. (1983). The stress-optic effect in optical fibers. J. Quantum Electron..

[51] Chan C.C., Shi C.Z., Jin W., Wang D.N. (2003). Improving the wavelength detection accuracy of FBG sensors using an ADALINE network. IEEE Photonics Technol. Lett..

[52] Caucheteur C., Chah K., Lhommé F., Blondel M., Mégret P. (2004). Autocorrelation demodulation technique for fiber Bragg grating sensor. IEEE Photonics Technol. Lett..

[53] Huang C., Jing W., Liu K., Zhang Y., Peng G.D. (2007). Demodulation of fiber Bragg grating sensor using cross-correlation algorithm. IEEE Photonics Technol. Lett..

[54] Negri L., Nied A., Kalinowsky H., Paterno A. (2011). Benchmark of peak detection algorithms in fiber Bragg grating interrogation and a new neural network for its performance improvement. Sensors.

[55] Lamberti A., Vanlanduit S., de Pauw B., Berghmans F. (2014). Influence of fiber Bragg grating spectrum degradation on the performance of sensor interrogation algorithms. Sensors.

[56] Yam L.H., Leung T.P., Xue K.Z., Wang B., Li D.B. Experimental study on modal strain analysis rectangula thin plates with holes. Proceedings of the 12th IMAC Conference.

[57] Kranjc T., Slavič J., Boltežar M. (2014). A comparison of the strain and the classic experimental modal analysis. J. Vib. Control.

[58] Bendat J.S., Piersol A.G. (2010). Random Data: Analysis and Measurement Procedures.

[59] DeMaCo Design for Manufacture of Composites. http://www.slc-lab.be/sites/default/files/DeMaCo%20Public%20report.pdf.

[60] Asco. http://www.asco.be/.

[61] Guillaume P., Verboven P., Vanlanduit S., Parloo E. Multisine excitations—New developments applications modal analysis. Proceedings of the 19th IMAC Conference.

[62] NI USB-6341. http://sine.ni.com/nips/cds/view/p/lang/en/nid/209069.

